# Research Trends of Artificial Intelligence in Lung Cancer: A Combined Approach of Analysis With Latent Dirichlet Allocation and HJ-Biplot Statistical Methods

**DOI:** 10.1155/pm/5911646

**Published:** 2024-12-04

**Authors:** Javier De La Hoz-M, Karime Montes-Escobar, Viorkis Pérez-Ortiz

**Affiliations:** ^1^Faculty of Engineering, Universidad del Magdalena, Santa Marta, Colombia; ^2^Departamento de Matemáticas y Estadística, Facultad de Ciencias Básicas, Universidad Técnica de Manabí, Portoviejo 130105, Ecuador; ^3^Facultad Ciencias de la Salud, Carrera de Medicina, Universidad Técnica de Manabí, Portoviejo 130105, Ecuador

**Keywords:** lung cancer, machine learning, topic modeling

## Abstract

Lung cancer (LC) remains one of the leading causes of cancer-related mortality worldwide. With recent technological advances, artificial intelligence (AI) has begun to play a crucial role in improving diagnostic and treatment methods. It is crucial to understand how AI has integrated into LC research and to identify the main areas of focus. The aim of the study was to provide an updated insight into the role of AI in LC research, analyzing evolving topics, geographical distribution, and contributions to journals. The study explores research trends in AI applied to LC through a novel approach combining latent Dirichlet allocation (LDA) topic modeling with the HJ-Biplot statistical technique. A growing interest in AI applications in LC oncology was observed, reflected in a significant increase in publications, especially after 2017, coinciding with the availability of computing resources. *Frontiers in Oncology* leads in publishing AI-related LC research, reflecting rigorous investigation in the field. Geographically, China and the United States lead in contributions, attributed to significant investment in R&D and corporate sector involvement. LDA analysis highlights key research areas such as pulmonary nodule detection, patient prognosis prediction, and clinical decision support systems, demonstrating the impact of AI in improving LC outcomes. DL and AI emerge as prominent trends, focusing on radiomics and feature selection, promising better decision-making in LC care. The increase in AI-driven research covers various topics, including data analysis methodologies, tumor characterization, and predictive methods, indicating a concerted effort to advance LC research. HJ-Biplot visualization reveals thematic clustering, illustrating temporal and geographical associations and highlighting the influence of high-impact journals and countries with advanced research capabilities. This multivariate approach offers insights into global collaboration dynamics and specialization, emphasizing the evolving role of AI in LC research and diagnosis.

## 1. Introduction

Lung cancer (LC), a leading cause of cancer-related death, is often diagnosed at an advanced stage, leading to poor survival rates [1]. It is primarily categorized into non–small cell lung cancer (NSCLC) and small cell lung cancer (SCLC), with distinct treatment approaches [2]. Early detection is crucial for improving survival rates, and imaging techniques are being explored for this purpose [3].

In recent years, artificial intelligence (AI) has emerged as a promising tool in the diagnosis and treatment of LC. Recent studies have highlighted the potential of AI algorithms to improve clinical outcomes, demonstrating a remarkable ability to detect and characterize lung nodules, as well as to predict treatment responses and guide therapy selection [4, 5]. However, although research on AI applied to LC is rapidly evolving, researchers face significant challenges in keeping up with the latest advances and identifying emerging trends in this field.

Several studies have employed scientific mappings, bibliometric approaches, and indicators to provide a comprehensive understanding of AI subfields applied to various types of tumors and to analyze trends within knowledge domains. In 2020, Salod and Singh [6] analyzed the use of machine learning (ML) in breast cancer prediction over a 5-year period, offering a review of methodologies and trends that inspire our approach. Their work highlights how AI can identify patterns in complex data, particularly within oncology, and helps position our study on AI in LC within the context of prior studies on other types of cancer.

Since 2021, studies such as those by Khairi et al. [7] on breast cancer have delved into the use of deep learning (DL) for histopathological image classification, demonstrating how this technology increases diagnostic precision and can be applied to the analysis of other types of cancer. This breast cancer-focused approach underscores the impact of DL in image analysis, a technique also relevant to LC diagnosis. [8]

In 2022, Musa et al. conducted a thematic analysis of the 100 most-cited articles on AI and ML in oncological research. This study reveals emerging topics and emphasizes the use of ML in optimizing oncology diagnostics and treatments. Musa et al.'s analysis provides a methodological basis for understanding how ML can be applied to LC, aligning our approach with these developments.

That same year, Shen et al. [9] explored the global use of AI in prostate cancer through a 22-year bibliometric analysis. Although focused on a different type of cancer, their research on keyword networks and coauthorship offers a methodological framework that we apply to our study and highlights similarities in the use of AI across oncology.

Recently, in 2024, Gencer [10] conducted a comprehensive analysis on the use of AI in LC, identifying key research areas and highlighting significant growth in publications, particularly in China and the United States. This analysis emphasizes the global context and the need for continued research in early LC detection using AI, providing a relevant perspective for our work.

Most analyses have primarily focused on specific subsets of AI applications, such as ML or DL, within particular types of tumors, like breast cancer and prostate cancer. Similarly, several recent studies have conducted comprehensive reviews of the literature on AI in LC, primarily using bibliometric analyses.

Moreover, a recent analysis by Corral, Borras, and Lievens [11] reviewed the utilization of radiotherapy in LC treatment in Europe, revealing considerable variability in the application of treatments such as stereotactic body radiotherapy (SBRT) and chemoradiotherapy (CRT) across different countries and patient populations. This study underscores the gap between optimal evidence-based utilization and actual clinical practice, emphasizing the need to improve adherence to therapeutic guidelines to ensure the best possible outcomes for LC patients.

However, despite the valuable insights provided by previous studies, none have employed advanced methodologies such as latent Dirichlet allocation (LDA) to analyze research trends in AI applied specifically to LC. LDA, which has proven effective in identifying research trends across various fields, including medicine, is being used for the first time in this study to analyze the existing literature on AI in LC. For instance, Duran-Ospina et al. [12] applied LDA in the field of infectious disease research to map complex areas in keratomycosis, demonstrating the method's capacity to uncover trends in intricate medical topics. Similarly, De La Hoz-M et al. [13] used LDA in COVID-19 research to capture complex biomedical data trends, highlighting its applicability to fields with vast and intricate datasets like oncology. Escobar et al. [14] employed LDA and HJ-Biplot in neuroendocrine tumor studies, showcasing the technique's utility in specialized medical fields with high data complexity.

This study is aimed at filling this gap by providing a comprehensive and up-to-date overview of research trends in the application of AI to LC, using advanced methodologies such as LDA [15] combined with the statistical technique HJ-Biplot [16]. This methodological combination will not only allow the identification of the main research topics and their evolution over time but also analyze the distribution of these topics across countries and scientific journals. The following key questions will guide this analysis:

Q1. What are the main research topics in this field?

Q2. How have these topics evolved over time?

Q3. What is the distribution of these topics among different countries and scientific journals?

By addressing these questions, this study is aimed at providing a deeper understanding of current research trends in AI in oncology, specifically in the context of LC, and at offering valuable guidance for future research in this rapidly evolving field.

## 2. Materials and Methods

### 2.1. Data Collection and Search Strategy

For data collection, we applied the Preferred Reporting Items for Systematic Reviews and Meta-analyses (PRISMA) protocol [17].

The data were gathered from two main databases commonly utilized by researchers: Scopus and Web of Science (WoS). These databases are widely employed across diverse scientific disciplines for literature searches and citation analysis, providing comprehensive coverage of scholarly literature, including journals, conference proceedings, patents, and other publications. Researchers frequently rely on these databases to find relevant literature, track citations, and assess the impact of their research [18].

Scopus is well-known for its extensive coverage of scientific disciplines, spanning natural sciences, social sciences, health sciences, and humanities, while WoS covers a broad spectrum of academic fields, including sciences, social sciences, arts, and humanities [18, 19]. Despite their individual strengths and weaknesses, researchers frequently integrate these databases to achieve comprehensive coverage of relevant literature for their studies [18, 19].

The merging of Scopus and WoS databases was achieved using the R “Bibliometrix” package and Excel, a method replicated from previous studies [20, 21].

Initially, separate searches were conducted (Table 1), and the data retrieved from these searches were subsequently merged during the second phase of the PRISMA protocol. The search strings employed were designed to encompass a broad range of keywords related to AI. Additionally, terms and keywords identified in previous bibliometric studies on AI applications in cancer were taken into account.

Only articles and reviews from scientific journals have been considered. After merging the results from Scopus and WoS and removing duplicates, a consolidated dataset of 7198 articles was obtained.

Once our consolidated dataset was established and the corresponding Excel file was created, we proceeded to eliminate articles that lacked abstracts or affiliation information. Through this process, a total of 89 articles were excluded from our analysis.

**Figure 1:**
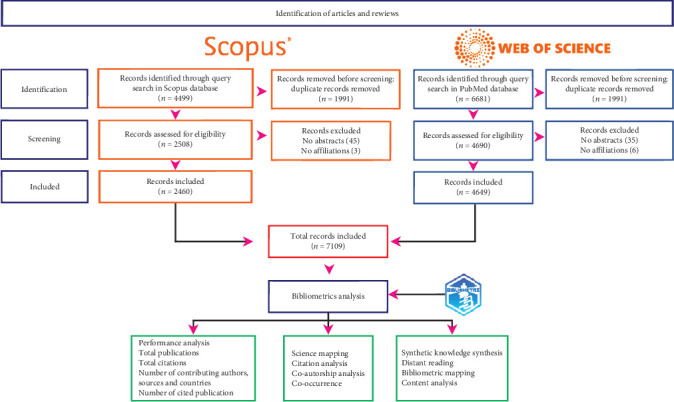
PRISMA diagram for inclusion of documents.

As a result, our dataset consists of 7109 articles. It is worth noting that some of these articles may be missing certain metadata. Nevertheless, essential data for subsequent analyses, such as title, abstract, publication year, and affiliation, were complete (Figure 1).

A critical aspect of ensuring robust results in any ML or topic modeling approach is the handling of missing data. In our study, we implemented a thorough process for managing incomplete records. Initially, we removed articles that lacked abstracts or essential metadata, such as title or publication year, which are crucial for topic modeling.

### 2.2. LDA

Topic modeling is a method in the field of natural language processing that falls under statistical latent semantic analysis. It involves statistically analyzing the distribution of terms within a corpus to uncover underlying topics or themes. LDA, introduced by Blei et al. in 2003 [15], is a probabilistic generative model of documents. It is a type of unsupervised ML algorithm capable of automatically discovering hidden or latent thematic structures, known as topics, and their probabilistic distributions across a large number of documents.

LDA assumes that a document is a mixture of several latent topics and that the words within the document were generated from these latent topics. The frequency of occurrence of words associated with a latent topic varies depending on the topic itself. In essence, words that are more relevant to a specific latent topic occur more frequently, thus representing the characteristics of each latent topic.

#### 2.2.1. Identifying Research Topics

The process of identifying topics through LDA was divided into three stages: (1) preprocessing, (2) construction of the LDA model, and (3) topic labeling. In the first two stages, we utilized LDAShiny [22], an open-source R package. LDAShiny offers a user-friendly web-based graphical interface for conducting scientific literature reviews. This package implements Bayesian approaches for LDA and ML algorithms.

##### 2.2.1.1. Preprocessing

Despite its apparent simplicity, the process involves several steps, including converting text to lowercase and removing punctuation marks, dashes, brackets, numbers, spaces, and other characters. Additionally, a standard list of words known as “stop-words” was identified and removed, as they primarily serve grammatical correctness, such as articles and prepositions. Consequently, the preprocessed data resulted in the creation of a document-term matrix. In this matrix, each document is represented as a vector containing an unordered collection of words. If the corpus contains a total of V words, each document becomes a V-dimensional vector, with the value of each element representing the frequency of the corresponding word in the document.

##### 2.2.1.2. Construction of the LDA Model

LDA assumes that topics (i.e., latent variables) are shared by all documents in the collection, but subject proportions vary stochastically between documents as they are randomly extracted from a Dirichlet distribution [23]. Establishing the optimal number of topics (*k*) for a given collection of items is not straightforward, as it requires balancing the need for a sufficient number of topics to cover all aspects within the document collection with the desire for a manageable number of topics that experts can easily understand and validate. To address this challenge, we generated LDA models with varying numbers of topics, ranging from 4 to 40. We conducted 1000 iterations for Gibbs sampling [24] and utilized default values for the Dirichlet parameters *α* and *β* from the topicmodel package [25].

Evaluation of LDA models involves various approaches. For instance, researchers have assessed topic models by measuring their accuracy in information retrieval tasks [26]. Additionally, statistical methods have been employed to gauge the predictive likelihood of a topic model in held-out documents, typically by computing perplexity [27]. In our study, we utilized a topic coherence measure known as CV [28], which is grounded in the distributional hypothesis [29] (Harris stated that words with similar meanings tend to co-occur in similar contexts). This coherence measure helps assess the semantic interpretability and coherence of the topics generated by the LDA model.

##### 2.2.1.3. Topic Labeling

Assigning semantic labels to the topics generated by the LDA model is crucial for providing a meaningful interpretation. While algorithmic analyses have limited capacity to comprehend the nuanced meanings of human language, manual labeling is considered a standard practice in topic modeling [30]. To ensure semantic accuracy, an expert in the field conducted the manual labeling of topics. This process involved integrating information from three key sources: the lists of most frequent words (most likely) provided by the model, as well as a sample of three document titles with their respective summaries classified by the algorithm.

#### 2.2.2. Quantitative Indices for Topic Characterization

LDA presents within its results, the topical-word probability distribution and the document-topical probability distribution. We used some of the indices proposed by Xiong et al. [31] given their usefulness, since it is not possible to determine the trends intuitively. These indices are obtained by aggregating topic-word and document-topic distributions at various levels.

The description of the index is as follows:

The distribution of topics over time is obtained by

(1) $$\begin{array}{c} \theta_{k}^{y}=\frac{\sum_{m\epsilon y}\theta_{mk}}{n^{y}} \end{array}$$

where *mϵy* represents articles published in a certain year, *θ*_*mk*_ is the proportion of the *k*th topic in each item, and *n*^*y*^ is the total number of articles published in the year.

To simplify the characterization of topics in relation to their trends, we employed straightforward regression slopes for each topic. In this analysis, the year served as the independent variable, while the proportions of topics within each corresponding year were treated as the dependent variable, following the approach outlined by Griffiths and Steyvers [32]. Topics exhibiting statistically significant positive (negative) regression slopes at a significance level of 0.01 were classified as increasing (declining) topics, respectively. The topics whose slopes did not reach statistical significance were classified as topics without trend or as fluctuating topics.

Topic distribution across countries is defined as the ratio of the *k*th topic in the country *c*.

(2) $$\begin{array}{c} \theta_{k}^{c}=\frac{\sum_{m\epsilon c}\theta_{mk}}{n^{c}}, \end{array}$$

where *mϵc* represents the articles in a particular country, *θ*_*mk*_ is the proportion of the *k*th topic on each item, and *n*^*c*^ is the total number of articles published in the country *c*.

Topic distribution across journals is defined as the ratio of the *k*th topic in the journal *j* : *θ*_*k*_^*j*^.

(3) $$\begin{array}{c} \theta_{k}^{j}=\frac{\sum_{m\epsilon j}\theta_{mk}}{n^{j}} \end{array}$$

where *mϵj* represents the articles in a particular journal, *θ*_*mk*_ is the proportion of the *k*th topic on each item, and *n*^*j*^  is the total number of articles published in the journal *j*.

Also, we defined *θ*_*k*_^*j*,*y*^ as the proportion of topics in the journal in a year.

(4) $$\begin{array}{c} \theta_{k}^{j,y}=\frac{\sum_{m\epsilon j\cap m\epsilon y}\theta_{\text{mk}}}{n^{j,y}} \end{array}$$

### 2.3. HJ-Biplot

Originally introduced by Gabriel [33], biplots serve as graphical representations of multivariate data, offering visualization of three or more variables, akin to a scatter plot illustrating the joint distribution of two variables. Gabriel's notable biplot factorizations include the GH-Biplot and JK-Biplot. The GH-Biplot focuses on exploring relationships among columns (variables) with superior representation quality, while the JK-Biplot assesses the similarity among rows (individuals) with emphasis on higher representation quality.

As an alternative to traditional biplots, the HJ-Biplot technique [21] has been introduced to simultaneously optimize the representation quality of both rows and columns within the same low-dimensional space.

Interpreting the HJ-Biplot requires integrating insights from various techniques, including multidimensional scaling, correspondence analysis, factor analysis, and classical biplots [21]. Distances between row markers convey their similarities, while the length of column markers (vectors) approximates the standard deviation. The cosines of the angles between column vectors serve as approximations of correlations: Acute angles indicate high positive correlation, obtuse angles indicate negative correlation, and right angles indicate uncorrelated variables. This analysis was conducted using the Multbiplot software [34].

## 3. Results

### 3.1. General Information of the Analyzed Dataset

**Figure 2:**
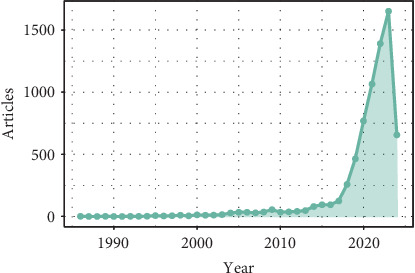
Annual scientific production about the collection of artificial intelligence in lung cancer from 7109 articles published between 1986 and April 2024.

Figure 2 shows a significant increase in the number of articles published on AI in LC over the years. Starting in 1986 with just one article, the number of publications (NPs) remained low in the following decades. However, from the 2010s onwards, there is an exponential growth in the number of articles, with a marked increase from 2014 onwards.

In particular, the most recent years, from 2018 to 2023, show a dramatic increase in the production of articles on this topic. In 2021 and 2022, the number of articles exceeds 1000, and in 2023, it reaches a peak of 1651.

These findings reflect a growing interest in the application of AI in LC research, highlighting the importance and potential impact of this area of study in the field of medicine and public health.

This research field was encompassed by a wide array of academic journals, totaling 1360, within the realm of AI research in LC, shedding light on the principal journals shaping this domain. Among them, “*Frontiers in Oncology*” (*Front. Oncol.*) stood out with 235 articles and a total citation count of 2999 since 2015. Similarly, “*Medical Physics*” (*Med. Phys.*) demonstrated significant contributions with 162 articles and 4540 total citations since 1997. “*Computers in Biology and Medicine*” (*Comput. Biol. Med.*) followed closely with 113 articles and 3128 citations since 2007 (Table 2).

Other notable journals include “*Cancers*” (*Cancers*) with 182 articles and 1958 citations since 2018 and “*European Radiology*” (*Eur. Radiol.*) with 79 articles and 1274 citations since 2007. “*Frontiers in Genetics*” (*Front. Genet.*) also made an impact with 61 articles and 437 citations since 2019. Moreover, “*PLoS ONE*” (*PLoS One*) demonstrated significant influence with 90 articles and 2068 citations since 2010, while “*IEEE Journal of Biomedical and Health Informatics*” (*IEEE J. Biomed. Health Inform.*) contributed with 64 articles and 1349 citations since 2015.

These journals, among others, have played pivotal roles in shaping the landscape of AI research in LC, reflecting the growing interest and importance of this field in the scientific community.

**Figure 3:**
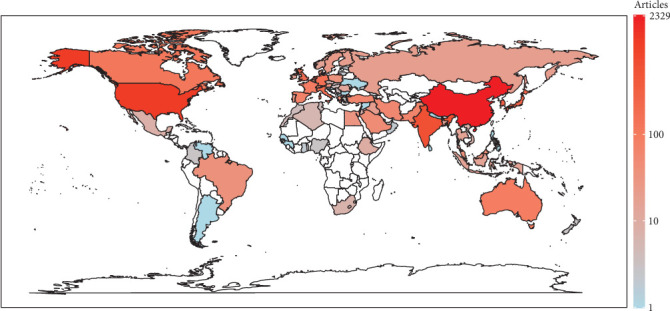
Distribution of geographical origins in the analysis of 7109 published articles on artificial intelligence in lung cancer between 1986 and April 2024.

The geographical diversity in research on AI applied to LC is striking, with contributions from 91 countries worldwide actively advancing knowledge in this field. China emerges as the leading country with the highest number of articles, totaling 2329, followed by the United States with 1216 articles. Remarkably, these two countries alone contribute nearly 50% of the total articles, amounting to 7109. India and Japan also rank among the top contributors, with 704 and 308 articles, respectively. Moreover, the United Kingdom, South Korea, Italy, and Germany demonstrate significant participation in research on AI in LC, each contributing hundreds of articles. Other countries such as the Netherlands, Canada, France, Spain, and Australia have also made notable contributions, further highlighting the widespread interest and importance of AI in LC research across borders and continents Figure 3.

### 3.2. LDA

The LDA model, optimized for coherence score, encompasses 28 topics. In Table 3, we present the 15 most probable terms (those with the highest probabilities) and their semantically related labels for each latent topic uncovered. Furthermore, we include the number of articles associated with each topic.

The Top 5 topics with the highest NPs are particularly noteworthy.

• Pulmonary nodule detection in CT scans (t_5): This topic focuses on the detection of pulmonary nodules in CT scans, a critical aspect for early diagnosis and treatment of LC. With 668 publications, it underscores the significance of AI in improving diagnostic accuracy.
• Patient survival and prognosis prediction (t_13): With 415 publications, this topic emphasizes the importance of predicting patient survival and prognosis in LC management. AI-driven prognostic models play a vital role in treatment planning and patient care.
• Clinical decision support systems (t_1): This topic, with 128 publications, highlights the development and implementation of clinical decision support systems in LC care. These systems aid healthcare providers in making informed decisions based on evidence and patient data.
• Radiation therapy planning and evaluation (t_24): With 381 publications, this topic underscores the role of AI in optimizing radiation therapy planning and evaluation for LC patients. AI techniques contribute to more precise treatment delivery and outcome assessment.
• Applications and developments in cancer research (t_27): This broad topic encompasses various AI applications and developments in LC research, with a significant focus on treatment strategies and technological advancements. With 630 publications, it reflects the diverse avenues of research in this field.

These findings underscore a robust interest in harnessing AI technologies across various facets of LC diagnosis, treatment, and research. The topics identified can be categorized into several overarching themes (Table 3):

• Clinical support and diagnostic technology: This theme encompasses a broad spectrum of topics, including clinical decision support systems, data collection, and image analysis techniques (t_1 clinical decision support systems, t_2 data collection and analysis in healthcare, t_3 methodology and frameworks for data analysis, t_4 AI in chest imaging, t_5 pulmonary nodule detection in CT scans, t6_image segmentation techniques, t_7 radiomic features extraction in medical imaging, and t_15 neural network architectures for imaging). Research within this area focuses on advancing clinical decision-making and diagnostic processes through technology-driven approaches. It involves the development of clinical decision support systems, efficient data collection methods, and innovative imaging techniques. These advancements are pivotal in enhancing patient care by equipping healthcare professionals with valuable insights and tools for precise diagnosis and treatment planning. Furthermore, studies concentrate on refining methodologies and frameworks for data analysis, ensuring the effective utilization of healthcare data to derive meaningful insights and guide clinical decision-making.
• Risk assessment and early diagnosis: Encompassing topics such as health risks, algorithm optimization, and biomarker identification (t_8 health risks and screening, t_9 algorithm optimization for image processing, t_11 early diagnosis and detection of cancer, t_12 diagnostic accuracy and specificity, t_17 biomarker identification and analysis, t_18 model performance and evaluation metrics, t_19 classification and predictive models, t_20 disease associations and predictive methods, t_21 breath analysis and sensor technology, t_22 immunotherapy response prediction, and t_26 model validation and testing). This theme emphasizes the significance of early detection and risk assessment in improving patient outcomes and survival rates for LC. Research in this domain explores various factors contributing to health risks, such as environmental exposures and genetic predispositions. It involves the development of algorithms for risk prediction and stratification. Additionally, studies focus on optimizing algorithms for image processing and analysis to enable early detection of pulmonary nodules and other cancerous lesions. Through the application of advanced AI techniques, researchers aim to enhance the accuracy and efficiency of diagnostic procedures, ultimately facilitating early intervention and improving patient prognosis.
• Treatment and therapy: covering topics related to tumor types, treatment response prediction, and therapy planning (t_10 tumor types and metastasis, t_13 patient survival and prognosis prediction, t_14 histological subtypes and tissue analysis, t_16 genetic mutations and drug response, t_23 DL models for medical imaging, t_24 radiation therapy planning and evaluation, t_25 ML algorithms for feature selection, t_27 applications and developments in cancer research, t_28 gene expression analysis in LC). Within this theme, researchers explore innovative approaches to personalized treatment and therapy planning for LC patients. Studies delve into tumor classification and metastasis patterns to inform treatment decisions and predict patient response to specific therapies. Moreover, research in this area focuses on developing predictive models for patient survival and treatment outcomes, enabling healthcare providers to tailor treatment regimens to individual patient needs. Additionally, advancements in radiation therapy planning and evaluation methodologies are investigated to optimize treatment delivery and minimize adverse effects. By integrating AI-driven technologies into treatment protocols, researchers aim to enhance treatment efficacy and improve patient quality of life while alleviating the burden of the disease.

Regression analysis was conducted to explore temporal trends in research topics related to the application of AI in LC. The results revealed diverse trajectories among the examined topics.

**Figure 4:**
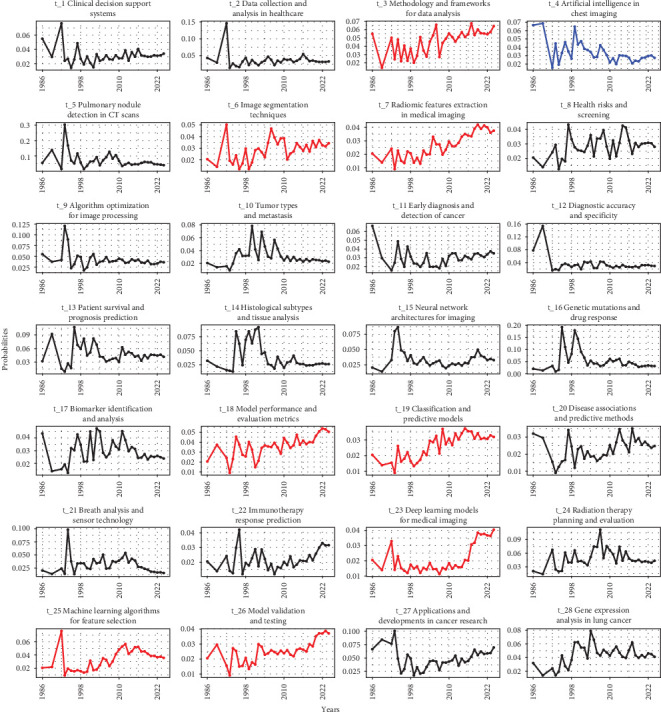
Topic trend.

Several topics demonstrated a significant upward trend in publications, signifying growing interest and research activity. Methodology and frameworks for data analysis (t_3) saw a surge in exploration, reflecting efforts to develop robust analytical approaches. Image segmentation techniques (t_6) experienced a significant uptick, reflecting innovations in extracting meaningful information from medical images. Radiomic feature extraction in medical imaging (t_7) garnered increased attention, indicating a shift towards leveraging advanced imaging techniques. Health risks and screening (t_8) witnessed a rise in interest, highlighting a proactive approach towards early detection and prevention. Tumor types and metastasis (t_10) experienced heightened exploration, underscoring efforts to understand cancer progression dynamics. Patient survival and prognosis prediction (t_13) saw increased research focus, reflecting endeavors to enhance patient outcomes through predictive analytics. Disease associations and predictive methods (t_20) garnered escalating interest, indicating strides in identifying risk factors and prognostic indicators. ML algorithms for feature selection (t_25) exhibited a notable surge, suggesting advancements in data-driven model optimization. Model validation and testing (t_26) witnessed increased scrutiny, emphasizing the importance of rigorous evaluation protocols. Gene expression analysis in LC (t_28) saw increased exploration, underscoring efforts to elucidate molecular mechanisms underlying cancer development (Figure 4).

Conversely, only AI in chest imaging (t_4) exhibited a declining trend in publications over time. This decline may signify a shift in focus towards more specific applications of AI in LC research, rather than a broad emphasis on chest imaging (Figure 4).

The rest of the topics did not demonstrate clear trends or exhibited fluctuations. These topics encompassed clinical decision support systems (t_1), data collection and analysis in healthcare (t_2), early diagnosis and detection of cancer (t_11), histological subtypes and tissue analysis (t_14), neural network architectures for imaging (t_15), genetic mutations and drug response (t_16), biomarker identification and analysis (t_17), model performance and evaluation metrics (t_18), breath analysis and sensor technology (t_21), immunotherapy response prediction (t_22), DL models for medical imaging (t_23), radiation therapy planning and evaluation (t_24), and applications and developments in cancer research (t_27). These topics either did not display pronounced trends or exhibited fluctuations in research activity over the analyzed period.

### 3.3. HJ-Biplot

**Figure 5:**
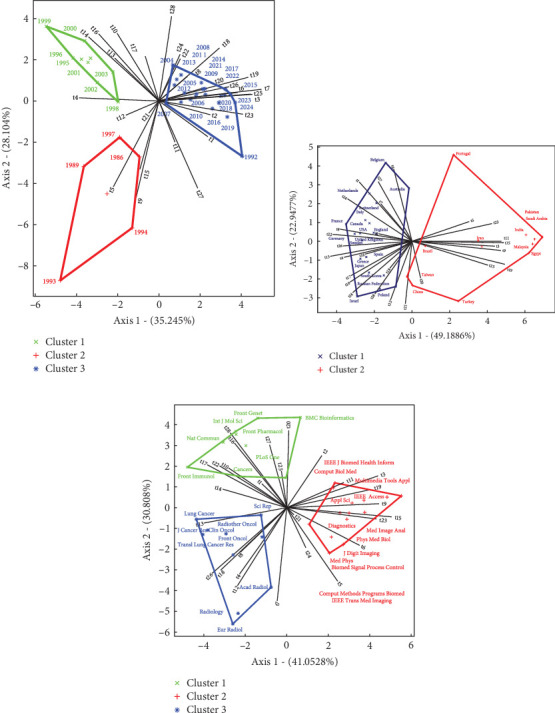
Association among (a) topics per year, (b) topics per country, and (c) topics per source using the HJ-Biplot method.

Figure 5 illustrates the distribution of the main topics among the Top 30 journals with the highest NPs on AI in LC.

Firstly, Figure 5(a) illustrates the years that are most closely associated with specific topics. As shown in the red cluster, the years from 1986 to 1997 are directly linked to topics such as t_12 (diagnostic accuracy and specificity), t_21 (breath analysis and sensor technology), t_5 (pulmonary nodule detection in CT scans), t_15 (neural network architectures for imaging), and t_9 (algorithm optimization for image processing). Following this, the green cluster reveals that the years 1998–2003 correlate with topics t_4 (AI in chest imaging), t_14 (histological subtypes and tissue analysis), t_16 (genetic mutations and drug response), t_10 (tumor types and metastasis), and t_17 (biomarker identification and analysis). Lastly, the red cluster in the same chart indicates that the years from 2004 to 2024 are directly related to a broad range of topics including t_28 (gene expression analysis in LC), t_24 (radiation therapy planning and evaluation), t_22 (immunotherapy response prediction), t_18 (model performance and evaluation metrics), t_8 (health risks and screening), t_20 (disease associations and predictive methods), t_19 (classification and predictive models), t_26 (model validation and testing), t6 (image segmentation techniques), t_7 (radiomic features extraction in medical imaging), t_25 (ML algorithms for feature selection), t_3 (methodology and frameworks for data analysis), t_23 (DL models for medical imaging), t_2 (data collection and analysis in healthcare), t_1 (clinical decision support systems), t_27 (applications and developments in cancer research), and t_11 (early diagnosis and detection of cancer).

Figure 5(b) delineates two distinct clusters representing the relationships between countries and research topics. The red cluster indicates that countries such as Portugal, Pakistan, Saudi Arabia, India, Iran, Malaysia, Egypt, Brazil, Taiwan, China, and Turkey are closely associated with topics t_5 (pulmonary nodule detection in CT scans), t_23 (DL models for medical imaging), t_11 (early diagnosis and detection of cancer), t_15 (neural network architectures for imaging), t_3 (methodology and frameworks for data analysis), t_9 (algorithm optimization for image processing), t_19 (classification and predictive models), t_25 (ML algorithms for feature selection), t_20 (disease associations and predictive methods), and t_21 (breath analysis and sensor technology). Conversely, the blue cluster includes countries such as Poland, Israel, the Russian Federation, South Korea, Japan, Greece, Spain, Sweden, Germany, the United Kingdom, England, the United States, Canada, France, Italy, Switzerland, Netherlands, Australia, and Belgium. These nations are prominently linked with topics t_6 (image segmentation techniques), t_27 (applications and developments in cancer research), t_7 (radiomic features extraction in medical imaging), t_1 (clinical decision support systems), t_24 (radiation therapy planning and evaluation), t_2 (data collection and analysis in healthcare), t_8 (health risks and screening), t_22 (immunotherapy response prediction), t_26 (model validation and testing), t_13 (patient survival and prognosis prediction), t_4 (AI in chest imaging), t_16 (Genetic mutations and drug response), t_17 (biomarker identification and analysis), t_10 (tumor types and metastasis), t_14 (histological subtypes and tissue analysis), t_18 (model performance and evaluation metrics), t2_8 (gene expression analysis in LC), and t_12 (diagnostic accuracy and specificity).

Finally, in Figure 5(c), the red cluster features journals such as *IEEE J. Biomed. Health Inform.*, *Comput. Biol. Med.*, *Multimedia Tools Appl.*, *IEEE Access*, *Appl. Sci., Diagnostics*, *Med. Image Anal.*, *Phys. Med. Biol.*, *J. Digit Imaging*, *Med. Phys.*, *Biomed. Signal Process Control, Comput Methods Programs Biomed.*, and *IEEE Trans. Med. Imaging*. These publications are closely associated with topics t_2 (data collection and analysis in healthcare), t_11 (early diagnosis and detection of cancer), t_3 (methodology and frameworks for data analysis), t_19 (classification and predictive models), t_9 (algorithm optimization for image processing), t_15 (neural network architectures for imaging), t_23 (DL models for medical imaging), t_6 (model validation and testing), t_21 (breath analysis and sensor technology), t_5 (pulmonary nodule detection in CT scans), and t_24 (radiation therapy planning and evaluation). The blue cluster includes journals such as *Eur. Radiol.*, *Radiology, Acad. Radiol.*, *Transl. Lung Cancer Res.*, *Front Oncol.*, *J. Cancer Res. Clin. Oncol.*, *Radiother. Oncol.*, *Lung Cancer*, and *Sci. Rep*. These are directly linked with topics t_7 (radiomic features extraction in medical imaging), t_12 (diagnostic accuracy and specificity), t_4 (AI in chest imaging), t_8 (health risks and screening), t_18 (model performance and evaluation metrics), t_26 (model validation and testing), and t_13 (patient survival and prognosis prediction). Lastly, the green cluster, comprising journals like *PLoS One*, *Cancers*, *Front. Immunol.*, *Nat. Commun.*, *Int. J. Mol. Sci.*, *Front. Pharmacol.*, *Front Genet*, and *BMC Bioinformatics*, is associated with t_14 (histological subtypes and tissue analysis), t_17 (biomarker identification and analysis), t_22 (immunotherapy response prediction), t_10 (tumor types and metastasis), t_1 (clinical decision support systems), t_16 (genetic mutations and drug response), t_28 (gene expression analysis in LC), t_27 (applications and developments in cancer research), t_20 (disease associations and predictive methods), and t_25 (ML algorithms for feature selection).

## 4. Discussion

The discussion is structured into several key sections to comprehensively address the findings of this study. First, the significant increase in publications on AI applied to LC after 2017 is analyzed, exploring the technological and computational factors that have contributed to this trend. Next, the geographical distribution of the research is examined, highlighting the predominance of China and the United States and the factors influencing this concentration, such as funding and international collaborations. Then, the characteristics and impact of the leading journals publishing research in this field are evaluated, including editorial policies and the types of studies they prioritize. Subsequently, the research topics identified through LDA analysis are discussed, analyzing their relevance to the diagnosis, treatment, and prognosis of LC, and how these topics interconnect and influence future research directions.

### 4.1. Temporal Trends

The growing interest in AI applications in LC oncology is evident in the significant increase in publications in this field. Research in this area has gained considerable momentum in recent years, as evidenced by the significant rise in the number of articles published over the past 5 years. Articles on AI and cancer were quite scarce until 2017, before showing a rapid increase thereafter. This trend coincided with the increased availability of computing resources and the growing popularity of AI solutions in other areas of healthcare [35]. This trend also became evident in certain oncology subspecialties. For instance, until 2017, there were only a limited number of articles related to AI and pediatric oncology, but this number increased significantly thereafter [10]. Additionally, researchers in the field of DL and breast cancer image classification began collaborating in 2017, and they have been publishing their work since then, with this field continuing to expand [8]. Therefore, it can be inferred that the growing global collaboration after 2017 facilitated the spread of awareness about AI in cancer research, resulting in a notable increase in productivity since that year.

The significant increase in publications on AI applied to LC research after 2017 can be attributed to several key factors. This period marked a turning point due to the convergence of technological advancements, enhanced computational resources, and a growing awareness of the potential of AI in cancer research.

The surge in AI-related publications in LC research after 2017 reflects the combined impact of technological advancements, enhanced computational capabilities, and a growing recognition of AI's transformative potential in medicine. These factors have created a fertile ground for innovation and discovery, leading to a marked increase in high-quality research aimed at improving cancer diagnosis and treatment.

### 4.2. Geographical Distribution

The global research landscape in AI applied to LC is dominated by China and the United States, accounting for nearly 65% of publications in this field [36]. This dominance could be due to substantial investments in research and development, advanced infrastructure, and strong collaboration networks in both countries In both China and the United States, governments have prioritized AI as a strategic component for their technological development, driving growth in research on medical applications, including oncology. In China, government initiatives such as the “New Generation Artificial Intelligence Development Plan” have provided significant support, while in the United States, robust funding from public agencies like the National Institutes of Health (NIH) and active participation from the private sector have been crucial [37]. The high level of corporate investment is a crucial factor for the success of R&D in these leading countries. The prolific scientific output in both countries is closely linked to the significant contribution of the corporate sectors to their R&D efforts [10].

The dominance of these two countries in AI research has also raised concerns about potential “cyber-colonization” and its impact on other regions of the world [37]. The high concentration of resources and scientific output in China and the United States could exacerbate disparities in access to advanced technologies and global health outcomes, particularly in regions with less developed AI infrastructure. This phenomenon highlights the need to foster more inclusive global collaborations to ensure that the benefits of AI in cancer research are distributed more equitably worldwide.

### 4.3. Journal Contributions

*Front. Oncol.* stands out as the most prolific journal in publishing research on AI applications in LC, as highlighted by Zhong et al. [36]. This finding is further supported by our own study, which also identifies *Front. Oncol.* as the leading venue for disseminating studies in this area. The journal consistently publishes key research in ML, DL, and classification techniques, areas that are crucial for advancing AI-driven solutions in LC diagnosis and treatment [36].

High-impact factors are a hallmark of the leading journals in this area, indicating their influence and the broad dissemination of the research they publish. Journals such as *Front. Oncol.*, and *Med. Phys.* consistently rank among the top in the field of oncology and medical physics, particularly in research related to AI and cancer. The high impact factor of these journals is reflective of their ability to attract high-quality manuscripts that are cited frequently, contributing to the advancement of both the scientific community and clinical practice-.

The editorial policies of these journals are designed to maintain rigorous scientific standards while also fostering innovation. Leading journals often require robust methodological approaches, thorough peer review, and clear demonstration of clinical relevance or potential impact on patient care. Leading medical journals are emphasizing the importance of rigorous clinical validation for AI technologies before their integration into practice [38]. These journals often require robust methodological approaches, thorough peer review, and clear demonstration of clinical relevance [39].

The types of research prioritized by these leading journals typically include studies that explore innovative AI applications with significant potential to improve LC outcomes. This includes research on AI-driven diagnostic tools, prognostic models, personalized treatment plans, and advancements in imaging techniques.

The leading journals in AI-related LC research are instrumental in shaping the trajectory of the field, with their editorial decisions and publication standards significantly influencing the direction and quality of scientific output. These journals are often distinguished by high-impact factors, strict editorial policies, and a clear emphasis on cutting-edge research that bridges technological advancements with clinical applications.

### 4.4. Research Topics

The application of LDA in AI research on LC has revealed key areas of interest and trends. Regarding topics with the highest NPs (pulmonary nodule detection in CT scans (t_5), patient survival and prognosis prediction (t_13), clinical decision support systems (t_1), radiation therapy planning and evaluation (t_24), and applications and developments in cancer research (t_27)), recent research has highlighted the significant impact of AI in improving LC outcomes. AI has been particularly effective in pulmonary nodule detection in CT scans, patient survival and prognosis prediction, clinical decision support systems, radiation therapy planning and evaluation, and applications in cancer research [40]. Notably, DL and AI have been identified as key trends in this field, with a focus on radiomics, convolutional neural networks, and feature selection [41]. These advancements have the potential to enhance the accuracy and efficiency of decision-making in LC care, particularly in prognosis and drug efficacy prediction [11]. However, the research trend in this area is still evolving, with a need for further exploration and development [42].

The surge of AI-driven research in LC is evident across various trending topics, including data analysis methodologies, image segmentation, radiomic feature extraction, health risks and screening, tumor types and metastasis, patient survival and prognosis prediction, disease associations and predictive methods, ML algorithms for feature selection, model validation and testing, and gene expression analysis (t_3, t_6, t_7, t_8, t_10, t_13, t_20, t_25, t_26, and t_28). These trends underscore a collective effort to advance LC research and improve patient outcomes through the application of AI-driven methodologies and technologies. Notably, studies have demonstrated the potential of AI in integrating radiomic, genomic, and clinical data for decision-making support in LC [43] and in predicting lymph node metastasis in CT images of T1 lung adenocarcinoma patients [44]. However, the application of ML techniques in daily clinical practice is still under exploration.

One of the key directions for future research lies in integrating data from multiple sources. For instance, the connection between image segmentation techniques (t_6) and radiomic feature extraction (t_7) suggests that future studies could focus on combining advanced image segmentation methods with radiomic analysis to develop more comprehensive diagnostic tools. These tools could provide not only precise tumor delineation but also detailed quantitative data that predict patient outcomes, potentially leading to earlier and more accurate diagnoses.

Another promising area is the development of multimodal AI models that incorporate data from various sources, including imaging, genomic, and clinical records. The intersection of gene expression analysis (t_28) with tumor types and metastasis (t_10) and patient survival and prognosis prediction (t_13) indicates that integrating genetic data with clinical and imaging information could result in more personalized treatment plans. Future research could explore how these multimodal models can be used to predict treatment responses and guide therapy decisions more effectively.

The optimization of ML algorithms is also a critical area for future research. The growing focus on feature selection (t_25) highlights the need to refine AI models by identifying the most relevant variables from complex datasets. Future studies could explore how advanced feature selection techniques can improve the accuracy and interpretability of AI models, particularly in predicting patient outcomes and treatment responses. Additionally, integrating these optimized models with robust validation and testing frameworks t_26) will be essential to ensure their reliability and safety in clinical practice.

Early detection and prevention strategies will continue to be a priority in LC research. The positive trend in health risks and screening (t_8) suggests that future research could focus on developing AI-driven tools that not only identify individuals at high risk of developing LC but also provide actionable insights for early intervention. Combining these screening tools with predictive methods (t_20) could lead to the creation of comprehensive prevention programs that are both more effective and more accessible.

Lastly, exploring the ethical and practical implications of AI integration in LC care will be a crucial area of future research. As AI technologies become more sophisticated and widespread, addressing issues such as data privacy, algorithmic bias, and the impact on clinical decision-making will be essential. Research in this area could focus on developing guidelines and best practices for the ethical use of AI in oncology, ensuring that these technologies are implemented in ways that benefit patients and healthcare providers alike.

### 4.5. Contribution to the Body of Knowledge

This study leverages the unique strengths of both LDA and HJ-Biplot to offer a comprehensive analysis of research trends in AI applications for LC. LDA, a powerful topic modeling technique, excels in uncovering latent themes within large datasets, allowing us to identify the primary research topics driving the field. Unlike traditional bibliometric methods, which often focus on citation metrics or coauthorship networks, LDA provides a more content-driven approach by examining the textual data directly to reveal underlying research themes. This enables a more nuanced understanding of the thematic evolution within the literature.

HJ-Biplot, on the other hand, adds a crucial dimension to the analysis by visually representing the complex relationships between these topics, geographical regions, and publication outlets. This technique allows for the simultaneous analysis of multiple variables, making it possible to identify patterns and clusters that would be difficult to discern using other methods. By integrating these two methods, our study not only identifies key research areas but also situates them within a broader global and temporal context, offering insights into how these topics are interconnected and how they have evolved across different regions and journals.

The combination of LDA and HJ-Biplot offers a novel perspective by providing a multidimensional analysis of research trends in AI applications for LC. This integrated approach allows us to go beyond simply identifying research topics; it enables us to explore how these topics are distributed globally, how they interact with one another, and how they are represented across different journals and countries. For instance, the HJ-Biplot visualization revealed distinct clusters of research activity, highlighting the dominance of certain countries like China and the United States in specific research areas such as pulmonary nodule detection and AI-driven prognostic models. These insights were not previously explored in the literature and demonstrate how certain regions are leading the charge in specific AI applications for LC, thereby guiding future research and collaboration efforts.

Moreover, the study identified emerging topics that are gaining prominence, such as the integration of AI with radiomics and genomics, which suggests a shift towards more personalized and data-driven approaches in LC care. These findings highlight the evolving nature of the field and provide a roadmap for future research that could focus on these burgeoning areas, further advancing the application of AI in oncology.

This study makes several significant contributions to the existing body of knowledge in the field of AI and LC research. First, it provides a comprehensive overview of the current research landscape, identifying key trends and areas of focus that are shaping the future of the field. By combining LDA with HJ-Biplot, the study offers a more detailed and contextualized analysis than previous studies, which often rely on more traditional bibliometric methods. This approach not only uncovers the thematic content of the literature but also provides insights into the geographical and temporal distribution of research, offering a global perspective on how AI is being applied to LC.

Additionally, the study's findings on emerging research topics and the identification of key contributing countries and journals provide valuable information for researchers and policymakers. These insights can inform decisions on where to allocate resources, how to foster international collaborations, and which areas of research may yield the most impactful results in the future. Overall, by advancing the methodology used to analyze research trends and offering new insights into the global distribution of AI research in LC, this study contributes meaningfully to the ongoing efforts to improve cancer diagnosis, treatment, and prognosis through the application of AI.

### 4.6. Research Limitations

While this study provides significant insights into research trends in AI applications for LC, several limitations must be acknowledged to contextualize the findings and guide future research.

Firstly, the study's reliance on data from major databases such as Scopus and WoS introduces a potential selection bias, as relevant studies published in other databases or nonindexed journals may have been excluded. This limitation could lead to an incomplete representation of the global research landscape, particularly in regions or institutions that publish outside these dominant platforms.

Secondly, the LDA model, while powerful, operates on the assumption that topics are independent and uniformly distributed across documents. This assumption may oversimplify the complex and interconnected nature of research themes, potentially obscuring nuanced overlaps and interdisciplinary connections within the AI and LC fields. Moreover, the topic labeling process in LDA, which requires manual interpretation, introduces a degree of subjectivity. Although efforts were made to ensure accuracy, this process might have led to biases, especially in categorizing more abstract or emerging research themes.

Additionally, while HJ-Biplot enhances the analysis by visualizing relationships between topics, geographical regions, and publication outlets, it may not capture the full complexity of the research landscape. The technique tends to highlight dominant patterns, which could overshadow less prevalent but potentially significant trends. This emphasis on the most prominent relationships might lead to an underrepresentation of emerging areas of research that are still developing traction.

Another limitation is the study's focus on published literature, which inherently excludes ongoing research and recent advancements that have not yet been formally published. In rapidly evolving fields like AI in LC, this time lag can affect the relevance and immediacy of the findings, as the study may not fully capture the latest developments.

Lastly, the integration of LDA and HJ-Biplot, while innovative, is not without its challenges. The effectiveness of this combination relies on the quality and completeness of the data, and any gaps or inconsistencies in the data could impact the robustness of the conclusions drawn.

In light of these limitations, future research should consider incorporating a broader range of data sources, including preprints and conference proceedings, to ensure a more comprehensive coverage of the field. Additionally, exploring alternative or complementary topic modeling techniques could provide a more dynamic and nuanced understanding of AI research trends in LC. By addressing these limitations, subsequent studies can build on the foundations laid by this research, further refining our understanding of the field and its global development.

## 5. Conclusions

This study provides a comprehensive overview of the research trends in AI applied to LC, employing a novel approach that combines LDA and the HJ-Biplot statistical technique. The findings highlight a significant increase in the volume of publications on AI in LC, particularly post-2017, underscoring the growing interest and recognition of AI's potential to enhance diagnostic and therapeutic outcomes in LC.

The analysis identified key research areas, such as pulmonary nodule detection, patient survival and prognosis prediction, clinical decision support systems, radiation therapy planning, and advancements in cancer research. These topics not only reflect the current focus of AI applications in LC but also reveal emerging trends, particularly in DL and radiomics, which are becoming increasingly integral to improving clinical decision-making and patient care in LC.

The geographical distribution of research contributions revealed that China and the United States are leading in this field, supported by significant investments in research and development. High-impact journals such as *Front. Oncol.* and *Med. Phys.* play a crucial role in disseminating advancements in AI applications for LC, further emphasizing the global collaborative efforts driving progress in this area.

Notably, this study is the first to employ LDA in analyzing research trends specific to AI in LC, providing a nuanced understanding of the thematic evolution and the distribution of research topics across countries and journals. The use of HJ-Biplot facilitated the visualization of complex relationships among these variables, offering insights into how specific topics have developed over time and geographically, and how they are represented in the literature.

## Figures and Tables

**Table 1:** Results of the bibliographic search in Scopus and Web of Science.

**Bibliographic database**	**Search data**	**Search string**	**Results**
Scopus	April 19, 2024	(TITLE-ABS-KEY (“AI”) OR TITLE-ABS-KEY (“artificial intelligence”) OR TITLE-ABS-KEY (“hybrid intelligent system⁣^∗^”) OR TITLE-ABS-KEY (“fuzzy expert system⁣^∗^”) OR TITLE-ABS-KEY (“machine intelligence”) OR TITLE-ABS-KEY (“artificial neural network⁣^∗^”) OR TITLE-ABS-KEY (“neural network”) OR TITLE-ABS-KEY (“transfer learning”) OR TITLE-ABS-KEY (“machine learning”) OR TITLE-ABS-KEY (“deep learning”)) AND (TITLE-ABS-KEY (“lung cancer”) OR TITLE-ABS-KEY (“lung neoplasm”) OR TITLE-ABS-KEY (“lung tumor”) OR TITLE-ABS-KEY (“lung carcinoma”)) (LIMIT-TO (DOCTYPE, “ar”) OR LIMIT-TO (DOCTYPE, “re”))	*N* = 6681
Web of Science	April 19, 2024	(TS = (“AI”) OR TS = (“artificial intelligence”) OR TS = (“hybrid intelligent system⁣^∗^”) OR TS = (“fuzzy expert system⁣^∗^”) OR TS = (“machine intelligence”) OR TS = (“artificial neural network⁣^∗^”) OR TS = (“neural network”) OR TS = (“transfer learning”) OR TS = (“machine learning”) OR TS = (“deep learning”)) AND (TS = (“lung cancer”) OR TS = (“lung neoplasm”) OR TS = (“lung tumor”) OR TS = (“lung carcinoma”)) AND DT = (Article OR Review)	*N* = 4499

**Table 2:** Top 30 scientific journals for research on artificial intelligence in lung cancer. TCs, total citations; NPs, number of publications; and PY_start, year of publication start. The table is organized in descending order by NP.

**Source**	**Abbreviation**	**NP**	**TC**	**PY_start**
*Frontiers in Oncology*	*Front. Oncol.*	235	2999	2015
*Cancers*	*Cancers*	182	1958	2018
*Medical Physics*	*Med. Phys.*	162	4540	1997
*Scientific Reports*	*Sci Rep*	148	3585	2014
*Computers in Biology and Medicine*	*Comput. Biol. Med.*	113	3128	2007
*Physics in Medicine and Biology*	*Phys. Med. Biol.*	100	2628	1999
*IEEE Access*	*IEEE Access*	100	1605	2017
*PLoS ONE*	*PLoS One*	90	2068	2010
*Diagnostics*	*Diagnostics*	82	568	2019
*European Radiology*	*Eur. Radiol.*	79	1274	2007
*Computer Methods and Programs in Biomedicine*	*Comput Methods Programs Biomed.*	71	1518	1986
*Biomedical Signal Processing and Control*	*Biomed. Signal Process Control*	71	744	2015
*IEEE Journal of Biomedical and Health Informatics*	*IEEE J. Biomed. Health Inform.*	64	1349	2015
*Frontiers in Genetics*	*Front. Genet.*	61	437	2019
*Frontiers in Immunology*	*Front Immunol.*	61	361	2021
*Multimedia Tools and Applications*	*Multimedia Tools Appl.*	55	288	2017
*Nature Communications*	*Nat. Commun.*	51	2774	2015
*Medical Image Analysis*	*Med. Image. Anal.*	50	4599	2005
*IEEE Transactions on Medical Imaging*	*IEEE Trans. Med. Imaging*	50	4773	1996
*Academic Radiology*	*Acad. Radiol.*	49	1184	2004
*Applied Sciences*	*Appl. Sci.*	44	331	2019
*Radiology*	*Radiology*	42	3040	1995
*Lung Cancer*	*Lung Cancer*	41	1116	1998
*BMC Bioinformatics*	*BMC Bioinformatics*	40	732	2004
*Journal of Cancer Research and Clinical Oncology*	*J. Cancer Res. Clin. Oncol.*	39	158	2005
*Radiotherapy and Oncology*	*Radiother. Oncol.*	37	1139	2006
*Translational Lung Cancer Research*	*Transl. Lung. Cancer Res.*	37	475	2017
*Journal of Digital Imaging*	*J. Digit Imaging*	36	860	1993
*Frontiers in Pharmacology*	*Front Pharmacol.*	36	156	2018
*International Journal of Molecular Sciences*	*Int. J. Mol. Sci.*	35	447	2017

**Table 3:** Topics discovered (T) from 7109 articles on AI in lung cancer published between 1986 and 2024. NPs, number of publications.

**T**	**Label**	**Top terms**	**NP**	**Themes**
t_1	Clinical decision support systems	studi, clinic, decis, report, includ, make, review, analysi, qualiti, literatur, trial, assess, provid, support, care	128	Clinical support and diagnostic technology
t_2	Data collection and analysis in healthcare	data, set, gener, inform, data_set, approach, real, medic, health, larg, time, sourc, collect, distribut, record	167	Clinical support and diagnostic technology
t_3	Methodology and frameworks for data analysis	method, propos, featur, multi, inform, dataset, effect, framework, show, experi, base, state, attent, problem, propos_method	495	Clinical support and diagnostic technology
t_4	Artificial intelligence in chest imaging	ai, intellig, artifici, artifici_intellig, intellig_ai, ci, case, chest, assist, conclus, report, softwar, algorithm, includ, assess	89	Clinical support and diagnostic technology
t_5	Pulmonary nodule detection in CT scans	nodul, detect, pulmonari, ct, pulmonari_nodul, comput, system, scan, posit, radiologist, malign, cad, sensit, aid, fals	668	Clinical support and diagnostic technology
t_6	Image segmentation techniques	segment, imag, method, manual, base, region, net, automat, similar, gener, ct, contour, autom, evalu, dice	234	Clinical support and diagnostic technology
t_7	Radiomic features extraction in medical imaging	featur, imag, ct, radiom, pet, extract, radiom_featur, ct_imag, tomographi, pet_ct, featur_extract, comput_tomographi, analysi, base, textur	316	Clinical support and diagnostic technology
t_8	Health risks and screening	screen, risk, low, year, mortal, high, popul, health, increas, rate, ag, low_dose, ldct, incid, effect	215	Risk assessment and early diagnosis
t_9	Algorithm optimization for image processing	imag, process, algorithm, techniqu, optim, propos, enhanc, pre, system, extract, paper, comput, work, filter, approach	253	Risk assessment and early diagnosis
t_10	Tumor types and metastasis	tumor, tissu, metastasi, primari, brain, metastas, breast, cancer, methyl, bone, type, site, metastat, dna, liver	100	Treatment and therapy
t_11	Early diagnosis and detection of cancer	diagnosi, earli, detect, stage, rate, diagnos, cancer, diseas, earli_stage, system, breast, lead, earli_detect, death, worldwid	150	Risk assessment and early diagnosis
t_12	Diagnostic accuracy and specificity	specif, sensit, diagnost, malign, sensit_specif, invas, lesion, adenocarcinoma, accuraci, benign, differenti, node, patholog, group, solid	149	Risk assessment and early diagnosis
t_13	Patient survival and prognosis prediction	patient, surviv, predict, risk, stage, treatment, prognost, factor, outcom, group, year, clinic, analysi, o, score	415	Treatment and therapy
t_14	Histological subtypes and tissue analysis	cell, nsclc, small, small_cell, carcinoma, cell_nsclc, subtyp, nsclc_patient, cell_carcinoma, squamou, adenocarcinoma, histolog, squamou_cell, tissu, sclc	118	Treatment and therapy
t_15	Neural network architectures for imaging	network, neural, neural_network, cnn, convolut, imag, convolut_neural, train, layer, architectur, network_cnn, propos, input, segment, ct_imag	154	Clinical support and diagnostic technology
t_16	Genetic mutations and drug response	mutat, drug, cell, egfr, target, activ, protein, effect, growth, line, resist, factor, statu, human, cell_line	370	Treatment and therapy
t_17	Biomarker identification and analysis	sampl, patient, group, biomark, control, marker, blood, tumor, healthi, mass, specif, diagnost, level, biopsi, identifi	153	Risk assessment and early diagnosis
t_18	Model performance and evaluation metrics	model, predict, auc, perform, curv, area, characterist, predict_model, oper, receiv, combin, base, evalu, receiv_oper, curv_auc	324	Risk assessment and early diagnosis
t_19	Classification and predictive models	classif, accuraci, classifi, dataset, perform, class, base, achiev, precis, ensembl, compar, method, approach, score, type	84	Risk assessment and early diagnosis
t_20	Disease associations and predictive methods	diseas, associ, mirna, predict, method, graph, similar, human, potenti, lncrna, relat, studi, mirna_diseas, valid, diseas_associ	120	Risk assessment and early diagnosis
t_21	Breath analysis and sensor technology	analysi, ann, neural, neural_network, artifici_neural, artifici, network, breath, compon, organ, discrimin, system, ga, voc, sensor	103	Risk assessment and early diagnosis
t_22	Immunotherapy response prediction	respons, immun, patient, treatment, therapi, tumor, pd, immunotherapi, predict, biomark, clinic, ici, efficaci, checkpoint, outcom	227	Risk assessment and early diagnosis
t_23	Deep learning models for medical imaging	learn, deep, deep_learn, imag, model, train, medic, dataset, base, learn_model, medic_imag, learn_base, covid, transfer, label	171	Treatment and therapy
t_24	Radiation therapy planning and evaluation	dose, plan, radiat, volum, patient, error, method, radiotherapi, reconstruct, time, imag, base, compar, treatment, evalu	381	Treatment and therapy
t_25	Machine learning algorithms for feature selection	machin, machin_learn, learn, featur, select, algorithm, method, random, support, svm, vector, featur_select, learn_algorithm, forest, random_forest	238	Treatment and therapy
t_26	Model validation and testing	valid, test, set, train, cohort, clinic, patient, develop, ci, extern, independ, score, test_set, conclus, intern	151	Risk Assessment and Early Diagnosis
t_27	Applications and developments in cancer research	applic, research, develop, clinic, approach, review, recent, challeng, technologi, potenti, advanc, treatment, provid, field, current	630	Treatment and Therapy
t_28	Gene expression analysis in lung cancer	gene, express, analysi, identifi, cell, genom, luad, relat, gene_express, high, cluster, pathwai, signatur, prognosi, subtyp	506	Treatment and Therapy

## Data Availability

The data that support the findings of this study are available from the corresponding author upon reasonable request.
